# Suicide among children and adolescents in India as a public mental health priority

**DOI:** 10.1007/s44192-026-00478-5

**Published:** 2026-10-21

**Authors:** Thangaduraipandi Ramakrishnan, Alexandra A. Deac, Shankavi Vivekanandhan, Tatiana Taylor Salisbury, Dhanasekara Pandian Ramasamy, Anish V. Cherian, Rajendra Kiragasur Madegowda, Nanda Kishore Kannuri

**Affiliations:** 1 Department of Psychiatric Social Work, National Institute of Mental Health and Neurosciences, Bengaluru, Karnataka 560029 India; 2 Institute for Connected Communities, University of East London, London, UK; 3 Centre for Global Mental Health, King’s College London, London, UK; 4 Department of Child and Adolescent Psychiatry, National Institute of Mental Health and Neuro Sciences, Bangalore, Karnataka 560029 India; 5 Department of Anthropology, University of Hyderabad, Hyderabad, Telangana 500046 India

**Keywords:** Children and adolescents, Suicide, Mental health, Psychosocial issues, Interventions

## Abstract

**Background:**

Suicide among children and adolescents (**≤** 18 years old) is a growing public health concern in India, contributing to an alarming 6% of total suicides reported in 2022. Despite policy efforts, systemic gaps in prevention, reporting, and mental health infrastructure persist.

**Methods/approach:**

This paper uses secondary data from the Accidental Deaths and Suicides in India (ADSI) reports (2014–2023) published by the National Crime Records Bureau (NCRB) to map the key trends and key drivers of suicide among youth in India. Descriptive report estimates on suicide data among individuals aged 0–18 years were categorised by gender, cause, and year. To interpret the underlying drivers of suicide, we applied the Socio-Ecological Model (SEM), which organises risk factors across four levels: individual, interpersonal, community, and societal.

**Results:**

Between 2014 and 2023, a total of 100,787 children died by suicide in India, with female children consistently outnumbering males. The most frequently reported causes were family-related problems (25,049), followed by academic failure (12,988), love affairs (12,988), and mental illness (6143). Nearly 29% of suicides were attributed to unknown or “other” causes, indicating gaps in documentation.

**Conclusion:**

Suicide among Indian children and adolescents is driven by multifaceted structural and psychosocial factors. A Bronfenbrenner’s socio-ecological approach reveals multiple points of intervention, including school-based mental health services, family support programs, anti-stigma initiatives, and community-level capacity building. A coordinated public health response tailored to each SEM level is essential to reverse current trends and safeguard child mental health.

## Highlights

Rising burden: Child and adolescent suicide in India is increasing, shaped by academic stress, family conflict, poverty, gender inequities, and digital harms.

Cultural context: Stigma, moral disapproval of suicide, and the trivialisation of children’s distress obscure underlying mental health drivers and limit help-seeking.

Policy–practice gap: Despite policies and programmes, services remain fragmented, urban-centred, and hampered by severe workforce shortages.

Ecological approach: Prevention must act across levels, individual, family, school, community, health systems, and policy, rather than through isolated interventions.

Future agenda: Co-produced, culturally relevant interventions and robust surveillance are essential to build resilience and reduce preventable deaths.

## Introduction

Suicide is a significant public health concern worldwide and a major cause of death among children and adolescents in low and middle-income countries [1]. Globally, approximately 46,000 children aged 10 to 19 die by suicide each year, translating to one child every 11 min [2]. India has the world’s largest young population, approximately 40% of the country’s total [2], witnessing one of the highest child (< 5–12 years old) and adolescent (< 13–18 years old) suicide rates globally [3, 4].

This alarming trend among children and adolescents underscores a crisis in making. Existing evidence suggests that risk factors are multifaceted and multi-layered, closely tied to contextual complexities and understandings of mental health and well-being, as well as the availability of support and services [3, 5–8]. Child suicide profoundly impacts families and communities, leading to long-term public health and economic consequences [9–11]. Each incident affects approximately 135 individuals, resulting in significant grief and trauma [12].

Informing holistic awareness-raising, prevention, and intervention strategies, transforming environments, and supporting children at all levels of society to prevent further tragedies remains a public health imperative. This special paper aims to discuss the issue of child and adolescent suicide rates in India, reported risk factors, and prevention strategies.

## Epidemiology of child and adolescent suicide in India

### Prevalence of child and adolescent suicide

Suicide among Indian youth (10–24 years old) is the second leading cause of death (Times of India, 2025), with over a 50% increase in the past two decades (National Crime Records Bureau [NCRB], 2019). Youth suicide rates are reported to be double that of the global average (25 vs. 13 per 100,000), with young women facing nearly six times higher suicide rates (25 vs. 4.1 per 100,000) [1].

Estimates suggest that more than 1.3 million children and adolescents have died by suicide in India in the past 26 years [3]. In 2022 alone, nearly 10,205 child and adolescent suicides were recorded in the country, marking a 27% increase compared to 2018 [13]. Notably, the suicide rate for this age group (per population) also rose, indicating that the increase is not solely due to population growth. The actual prevalences are thought to be 25% higher [8, 14]. Table 1 summarises the reported suicide deaths of minors from 2014 to 2022 as per NCRB’s annual Accidental Deaths and Suicides in India reports. Although national suicide statistics do not account for disaggregated cultural groups, suicide attempts, or suicidal ideation across different communities and groups in India, this data points to an acute mental health crisis within the country [15].

**Table 1:** Death by suicide among children (0–18 years) in India from 2014 to 2023 (NCRB, 2014–2023)

S.no	Year	Male	Female	Transgender*	Total
1	2014	5581(51%)	5369 (49%)	0	10,950
2	2015	4462 (47%)	4946 (53%)	0	9408
3	2016	4187 (46.7)	4764 (53.2%)	0	8951
4	2017	4350 (46.6%)	4966 (53.3%)	0	9316
5	2018	4439(47%)	4992 (53%)	0	9431
6	2019	4405 (46%)	5208 (54%)	0	9613
7	2020	5392 (47.3%)	6004 (52.6%)	0	11,396
8	2021	5075 (47.2%)	5655 (52.6%)	2 (0.01%)	10,732
9	2022	4616 (45.2%)	5588 (54.75%)	1 (0.00%)	10,205
10	2023	4961 (46.03%)	5823 (53.98%)	1 (0.00%)	10,785
**Total**		47,468 (47.11%)	53,315 (52.89%)	4 (0.003%)	100,787

*Transgender rates were not reported between 2014–2020

Data source: Accidental Deaths and Suicides in India (ADSI, 2014–2023)

**Research objectives**

1. Analyse Trends and Causes: Examine patterns of child suicides from 2014 to 2023 using comprehensive NCRB data to understand the scope and evolution of this crisis.
2. Map Key Drivers: Utilise the Socio-Ecological Model (SEM) to identify multi-level risk factors spanning individual, interpersonal, community, and societal dimensions.
3. Identify Intervention Points: Determine evidence-based opportunities for policy development and clinical practice to prevent future tragedies.

## Methodology

### Data source

The study utilised secondary data obtained from the Accidental Deaths and Suicides in India (ADSI) reports published annually by the National Crime Records Bureau (NCRB). ADSI is the most comprehensive national-level database that systematically compiles information on mortality due to accidents and suicides across all states and union territories in India.

For this study, data covering a ten-year period from 2014 to 2023 were extracted (ref here). Data source link: https://www.ncrb.gov.in/accidental-deaths-suicides-in-india-adsi.html.

### Study population

The analysis focused on children and adolescents aged 0–18 years. This age range reflects the NCRB classification system, which categorises suicide deaths for individuals below 18 years and provides age-group disaggregation for minors. Data were extracted for all states and union territories to allow comprehensive coverage and comparison across geographical regions.

### Analytical framework: socio-ecological model

The study employed the Socio-Ecological Model (SEM) as the guiding analytical framework to interpret patterns and determinants of suicide among children and adolescents. The SEM conceptualises suicide as the outcome of interacting factors operating at multiple levels:

Individual level: age, gender, personal vulnerabilities, mental health-related factors.

Interpersonal level: family dynamics, parental conflict, relationships with peers and teachers.

Community level: school environment, neighbourhood influences, and community support systems.

Societal level: socio-economic conditions, cultural norms, media portrayal, and state-level factors.

Using the SEM allowed for a structured categorisation of the risk factors identified in the ADSI dataset. It also facilitated a holistic interpretation of suicide patterns by recognising that individual behaviours are shaped by broader social and structural determinants.

## Data extraction

Relevant variables were extracted from ADSI reports, including age-group classification, gender, causes of suicide, state/UT-wise incidence. Data were tabulated year-wise and categorized according to SEM levels. Any inconsistencies or missing data in the ADSI reports were noted and addressed through cross-verification with annual NCRB summaries.

## Analytical approach

A descriptive analytical approach was adopted. Trend analysis was conducted to examine changes in suicide incidence over the ten-year period. Causes of suicide were grouped based on the SEM categories to understand the distribution of risk factors across ecological levels. State-level comparisons were undertaken to highlight variations in suicide patterns across different geographical regions.

## Result

A total of 90,002 child suicides were recorded between 2014 and 2023. Of these, 53,315 (52.76%) were female, 47,468 (47.22%) were male, and 4 (0.004%) were transgender. Reported suicide cases climbed from roughly 8000 in 2014 to over 10,000 in 2020 and 2023, with a slight dip estimated in 2023. This represents an increase of well over 25% in less than a decade. Of note is that suicide attempts were illegal before 2017 [16], and the cause of death attribution might have been misclassified or under-reported [17–19].

Another striking pattern is the gender distribution observed in Table 1. In 2021, for example, NCRB recorded 5655 female and 5077 male child suicide deaths (plus a negligible number of transgender cases). This female predominance among minors, has been consistently observed since 2015 [20]. Adolescent girls often face vulnerabilities stemming from gender-based disadvantages, including higher risks of coerced marriages, early pregnancies, and restrictive social norms [21–23]. However, recent evidence suggests that gender-specific risks are increasingly complex. While girls are frequently highlighted in this context, adolescent boys in India also face significant vulnerabilities, particularly regarding sexual abuse and severe barriers to help-seeking due to social stigma. These varied forms of trauma and coercion, regardless of gender, contribute significantly to hopelessness and suicide risk in the absence of robust support systems.

## Reported causes of suicide among children and adolescents and contextual drivers

Child and adolescent suicides in India stem from a complex interaction of individual vulnerabilities and environmental stressors [24]. Between 2014 and 2023, nearly 100,787 suicides were reported in those under 18 years (Table 2 below).

**Table 2:** Reported causes of suicide among children and adolescents (< 18 years old) (NCRB 2014–2023)

Year	2014	2015	2016	2017	2018	2019	2020	2021	2022	2023	Total
Marriage related issues	307	196	221	186	298	197	158	194	206	253	2216
Failure in examination	1284	1360	1330	1489	1529	1577	1129	864	1123	1303	12,988
Family problems	1586	2139	2006	2251	2236	2468	4006	3233	2556	2568	25,049
Mental illness	422	522	411	599	492	553	823	822	749	750	6143
Other prolonged illness	671	356	498	490	410	335	476	557	506	456	4755
Love affairs	899	814	942	1006	1131	1297	1337	1495	1578	1724	12,223
Causes not known	1815	1280	1412	1431	1361	1311	1421	1336	1228	1406	14,001
Other causes	3563	2208	1672	1384	1579	1404	1529	1488	1627	1822	18,276
Total	10,547	8875	8492	8836	9036	9142	10,879	9989	9573	10,282	95,651

Source: Accidental Deaths and Suicides in India (ADSI, 2014–2022)

**Table 3:** Ecological framework for child and adolescent suicide prevention in India

Level	Areas of action
Child/adolescent (individual)	Life-skills education (resilience, problem-solving, stress tolerance); promotion of help-seeking; access to age-appropriate counselling, including digital interventions.
Family/peers	Parent and caregiver training in recognition of distress; family counselling to strengthen communication; structured peer-support programmes.
School/community	Integration of mental health into curricula; deployment of trained school counsellors/psychologists; gatekeeper training for teachers; engagement of parents and community stakeholders through awareness workshops and anti-stigma campaigns.
Health system	Strengthening of District Mental Health Programme (DMHP) with child- and adolescent-focused services; crisis protocols linking schools, primary care, and specialist care; expansion of Tele-MANAS and digital platforms.
Policy/society	Development of a national suicide surveillance and response system; adoption of a dedicated child and adolescent mental health policy with sustainable financing; establishment of accessible emergency support lines (including digital and text-based platforms); enforcement of responsible media reporting guidelines; and sustained implementation of the National Suicide Prevention Strategy (2022) aligned with WHO’s LIVE LIFE framework.

The drivers of suicide in this age group span multiple domains. The most common categories were family problems (25,049), “exam failure” (12,988), and “love affairs” (12,223) [24]. Academic pressure is a salient determinant, with examination failure emerging as the leading specifically recorded cause, reflecting the weight of India’s competitive educational culture [25]. Family and interpersonal stressors, conflict, neglect, abuse, and relationship crises further contribute, particularly affecting young women [8].

Psychological vulnerabilities such as depression, suicidal ideation, stress, and impulsivity are prominent, but care-seeking remains limited [26]. Strikingly, “Causes Not Known” (14,001) and “Other Causes” (18,276) together account for more than five times the number of suicides attributed directly to mental illness (6143). This pattern does not suggest that mental health is unimportant; rather, it reflects systemic under-recognition and stigma. In India, child and adolescent distress is often trivialised as “naïve” or “immature,” while suicide itself is viewed as morally unacceptable [27]. Families and officials may therefore classify deaths into vague categories instead of acknowledging psychological suffering [14, 19].

Other known risk factors of suicide among children and adolescents include socioeconomic hardship, digital risks, and cultural factors such as stigma, gendered expectations, technology-facilitated gender-based violence, and discrimination, which compound vulnerability, while remaining poorly captured in surveillance [20, 28, 29]. Together, these domains underline the multifactorial nature of suicide and the need for comprehensive, systems-level prevention strategies (Table 3).

## Current policies, interventions and gaps

India has made notable advances in mental health policy and legislation. Most significantly. the Mental Healthcare Act (2017) effectively superseded Sect.  309 of the Indian Penal Code, which had previously criminalised suicide attempts, a colonial-era remnant that long hindered help-seeking. This Act enshrined the right to mental healthcare [17], while the National Mental Health Policy (2014) and District Mental Health Programme (DMHP) expanded decentralised care. Adolescent health promotion has been pursued through the Rashtriya Kishore Swasthya Karyakram (RKSK, 2014) and the School Health Programme under Ayushman Bharat (2018). More recently, the National Suicide Prevention Strategy (2022) explicitly aligned with WHO’s LIVE LIFE framework, aiming to reduce suicide mortality by 10% by 2030 (WHO, 2021).

Alongside these policies, several service innovations have emerged. Tele-MANAS, launched in 2022, expanded access to counselling and crisis support, while Manodarpan provides school-based psychological support. The SURAKSHA Project in Karnataka, piloting community suicide surveillance and structured post-attempt care aligned with LIVE LIFE, represents a promising model but remains limited in scale.

Despite these advances, significant gaps remain. Child- and adolescent-specific provisions are rarely prioritised, financing is inadequate, and stigma continues to deter help-seeking. Crucially, India faces an acute workforce shortage—fewer than 0.3 psychiatrists per 100,000 population, with even fewer trained psychologists, psychiatric social workers and school counsellors [28, 30]. Surveillance systems under-report suicides and attempts, restricting the evidence base for planning. The result is a fragmented landscape in which promising initiatives fail to translate into large-scale, child-centred suicide prevention. Yet the challenge extends beyond clinical capacity. Evidence increasingly highlights the need for multisectoral approaches that integrate health, education, social services, legal systems, and community structures. Teachers, community health workers, and local governance institutions often serve as first points of contact for young people experiencing distress, particularly in low-resource settings .

## Ecological framework for prevention

The rising burden of suicide among children and adolescents in India requires a comprehensive, multi-sectoral strategy. To address individual and social gaps and support innovative systems that centre the voices and needs of children and adolescents, an ecological approach is needed. Bronfenbrenner’s ecological systems theory (1979) posits that individuals are shaped by complex interactions within the environment in which they live. This framework positions the child within nested environments, including family, peers, schools, communities, health systems, and broader policy structures. By mapping risk and protection across levels, the framework highlights that no single intervention is sufficient: prevention must integrate supports across the ecological spectrum, from life-skills training and family counselling to school-based mental health services, community surveillance, and national policy reform.

## Recommendations

### Promotion and awareness

Schools are key settings for prevention. Integrating life-skills training into curricula builds resilience and stress tolerance, while awareness programmes for students, parents, and teachers can reduce stigma and encourage help-seeking [31, 32]. Gatekeeper training equips teachers and caregivers to identify early warning signs and act appropriately [33].

### Identification and treatment

Systematic screening protocols in schools and primary care can enable early detection of distress [34]. Evidence-based therapies, such as cognitive-behavioural and family interventions, have shown effectiveness in reducing suicidal behaviours [35, 36]. Digital platforms like Tele-MANAS and online CBT can extend reach in contexts of workforce shortages [37, 38].

### Restricting access and surveillance

Reducing access to lethal means remains a priority. Bans on highly hazardous pesticides have already contributed to declines in self-poisoning and should be expanded and enforced [39]. Parallel efforts should strengthen suicide surveillance, as piloted in the SURAKSHA Project, which combines community monitoring and post-attempt support aligned with the WHO’s LIVE LIFE framework [40].

### Support systems

Families, peers, and educators affected by suicide attempts or deaths require structured postvention, including grief counselling and follow-up care, to mitigate trauma and prevent contagion [41]. Schools should establish crisis response protocols to provide coordinated support in such situations.

### Human resources and policy reform

India has fewer than 0.3 psychiatrists per 100,000 population, and most are concentrated in urban centres [28]. For families in rural areas, reaching a specialist often requires travelling long distances at significant financial cost, frequently involving loss of wages and disruption to schooling. The journey itself can be arduous and, for many, prohibitive, leading to delayed or foregone care. This inequitable distribution leaves rural children particularly vulnerable, with primary care providers often the only available point of contact. Strengthening community-based services, training non-specialist health workers, and expanding digital platforms such as Tele-MANAS are therefore essential to bridge gaps in access and reduce the perilous burden of travel for care.

### Future research

Despite promising initiatives, evidence on what works for preventing child and adolescent suicide in India remains limited. Future studies should prioritise the co-production of culturally relevant interventions, designed with children, adolescents, families, and educators. Rigorous evaluation of school-based models, family-focused programmes, and digital platforms is particularly needed in rural and resource-poor settings.

Strengthening longitudinal surveillance and self-harm registries will also help to identify trajectories of risk and resilience across adolescence. Such systems could help identify and monitor high-risk subgroups within the adolescent population, including LGBTQIA+ youth, adolescents living with mental health or substance use disorders, and young people growing up in contexts of heightened vulnerability such as parental incarceration, sex work, or domestic violence. Generating inclusive, equity-focused evidence on these groups will be essential for informing scalable and sustainable strategies.

## Conclusion

Child and adolescent suicide in India represents a pressing public mental health crisis, with rates rising despite existing policies and programmes. The drivers are multifaceted, ranging from academic pressure and family conflict to poverty, stigma, and limited access to care, underscoring the need for coordinated, system-level responses. Current interventions remain fragmented, with critical gaps in child-focused services, workforce capacity, and community-based surveillance.

Strengthening prevention requires an ecological approach that integrates resilience promotion, early identification, accessible treatment, postvention, and sustained policy reform. Priorities include embedding counsellors in schools, expanding family- and community-based interventions, enforcing means restriction, and addressing the stark shortage and maldistribution of mental health professionals. Digital platforms such as Tele-MANAS, combined with community gatekeeper training and surveillance models like SURAKSHA, offer scalable solutions, particularly for rural populations.

Future efforts must emphasise co-produced, culturally relevant interventions that empower children, adolescents, parents, and educators to recognise and respond to distress. By investing in such approaches, India can move towards reducing preventable deaths and creating supportive environments that allow young people to thrive.

## Data Availability

This study utilized the secondary data extracted from NCRB-ADSI https://www.ncrb.gov.in/accidental-deaths-suicides-in-india-adsi.html.
